# Clozapine-Induced Late Agranulocytosis and Severe Neutropenia Complicated with *Streptococcus pneumonia*, Venous Thromboembolism, and Allergic Vasculitis in Treatment-Resistant Female Psychosis

**DOI:** 10.1155/2015/703218

**Published:** 2015-02-10

**Authors:** Christina Voulgari, Raphael Giannas, Georgios Paterakis, Anna Kanellou, Nikolaos Anagnostopoulos, Stamata Pagoni

**Affiliations:** ^1^3rd Department of Internal Medicine, Athens General Regional Hospital “G. Gennimatas”, Mesogeion Avenue 154, 115 27 Athens, Greece; ^2^Flow Cytometry Laboratory, Department of Immunology, Athens General Regional Hospital “G. Gennimatas”, Athens, Greece; ^3^Department of Hematology, Athens General Regional Hospital “G. Gennimatas”, Athens, Greece

## Abstract

Clozapine is a second-generation antipsychotic agent from the benzodiazepine group indicated for treatment-resistant schizophrenia and other psychotic conditions. Using clozapine earlier on once a case appears to be refractory limits both social and personal morbidity of chronic psychosis. However treatment with second-generation antipsychotics is often complicated by adverse effects. We present a case of a 33-year-old Caucasian woman with a 25-year history of refractory psychotic mania after switching to a 2-year clozapine therapy. She presented clozapine-induced absolute neutropenia, agranulocytosis, which were complicated by *Streptococcus pneumonia* and sepsis. Clozapine-induced thromboembolism of the common femoral and right proximal iliac vein, as well as allergic vasculitis, was diagnosed. She achieved full remission on granulocyte-colony stimulating factor and specific antibiotic treatment. Early detection of severe clozapine-induced absolute neutropenia and agranulocytosis enabled the effective treatment of two among its most severe complications. Additional evidence to the previously reported possible causal relation between clozapine and venous thromboembolism is offered. Finally, clozapine-induced allergic vasculitis is confirmed as a late adverse effect of clozapine therapy.

## 1. Introduction

Clozapine is an atypical antipsychotic agent, also known as neuroleptic, from the dibenzodiazepine group. Benefits of being on this drug can include relief from both positive and negative symptoms in patients with schizophrenia. Clozapine is often called an “antimanic agent” and remains the most effective drug for patients with affective psychotic mania, who fail to respond to adequate trials with two antipsychotics [1]. It is also the antipsychotic of choice for refractory schizophrenia [2], with advantages in terms of burden of psychiatric symptoms, avoidance of hospitalization, potential for social and occupational rehabilitation [2, 3], and healthcare costs [4]. Clozapine also reduces suicidal behavior in schizophrenia [5].

These beneficial effects of clozapine portrait in patients with schizophrenia are also observed in patients with bipolar disorder (BD) [6]. Clozapine treats acute manic/hypomanic/mixed and depressive episodes, prevents further episodes, and does not aggravate the disorder or its comorbidities [7, 8]. As BD is the 6th leading cause of disability-adjusted life years among people between 15 and 44 years of age [9] and untreated BD is associated with substantial morbidity and mortality, effective treatment is crucial [10].

Lately in patients with BD, when other mood stabilizers including lithium, valproate, carbamazepine, lamotrigine, and oxcarbazepine fail, switching to clozapine can be tried. According to the American Psychiatric Association (2006), switching must be evidence-based and take into account status of the condition being treated, efficacy, side effect profile, potential drug-to-drug interactions, required laboratory monitoring, cost of the drug, and patient's pregnancy status or plan.

Clozapine acceptance by patients and the frequency of its prescribing by clinicians have been hampered by apprehension regarding adverse effects with life-threatening potential, markedly significant agranulocytosis and neutropenia [11, 12]. Benign neutropenia occurs more frequently than agranulocytosis. Transient neutropenia, in which the neutrophil count drops below a defined value but returns to normal values with continued clozapine treatment, occurs frequently [13]. Patients may also display a diurnal variation in the number of circulating neutrophils, such that a morning pseudo-neutropenia was normalized in the afternoon [13].

The most serious side-effect of clozapine is agranulocytosis, and therefore in view of this risk, all patients receiving clozapine undergo regular white cell count monitoring, which should be carried out every week for the first 18 weeks of treatment. Agranulocytosis is defined as a drop in absolute neutrophil count <500 per cubic millimeter and can be fatal in the presence of an infection. Clozapine is immediately discontinued if a patient develops leucopenia (white cell count <3.06 ∗ 10^9^ per liter with a satisfactory neutrophil count) or neutropenia (neutrophil count <1.56 ∗ 10^9^ per liter) and further treatment with the drug is subsequently contraindicated.

Annually, the incidence of drug-induced agranulocytosis, excluding cytotoxic agents, is estimated to be approximately seven cases per one million people [14]. The mortality rate from drug-induced agranulocytosis is approximately 5 to 10 percent but decreases with early identification and treatment [15]. Among the antipsychotics, clozapine displays the highest propensity to induce agranulocytosis predominantly occurring in the first year of treatment [14, 15]. Nevertheless, sudden late onset of blood dyscrasia has been reported [16].

We describe herein a patient with BD after switching to a 2-year clozapine therapy. She presented clozapine-induced absolute neutropenia and agranulocytosis, which were complicated by* Streptococcus pneumonia* and sepsis. Moreover, clozapine-induced thromboembolism of the common femoral and right proximal iliac vein, as well as allergic vasculitis, was diagnosed upon Emergency Department presentation and during hospitalization. She achieved full remission on granulocyte-colony stimulating factor (G-CSF) and specific antibiotic treatment.

## 2. Case Presentation

A 33-year-old Caucasian woman presented to the Emergency Department of our hospital with a 4-day unilateral right leg peripheral edema and a one-week continuous fever associated with a productive cough. For these symptoms, she was receiving clarithromycin 500 mg b.i.d. and paracetamol 500 mg t.i.d. for the previous 6 days. The patient had a 10-year history of BD and she obtained full remission of psychotic symptoms with a 2-year clozapine (200 mg b.i.d.) treatment. She was also receiving levothyroxine sodium (125 *μ*g QD) for hypothyroidism.

The patient was unmarried and lived with her parents and 2 years before she had been admitted to the outpatient unit of the psychiatric clinic of our hospital with behavioral disorders of increasing severity. She presented outwardly aggressiveness, both verbally and physically, with manifested episodes of psychomotor agitation and rage. These symptoms were often exchanged with the patient becoming increasingly withdrawn and apathetic, with emotional blunting, pathological quilt, and remarkable anhedonia and anergy. She complained of decreased need for sleep, while during examination she was more talkative than usual, kept talking, and was easily distracted. According to the DSM-IV criteria for schizoaffective disorder, the patient was diagnosed with a mixed episode of bipolar schizoaffective disorder and was switched from quetiapine fumarate (100 mg QD) to clozapine.

With clozapine titration (initiated at a dose of 12.5 mg/day and gradually titrated to 100 mg QD), significant improvement in delusional ideation and behavioral disorder was reported from the first months of treatment. Upon clozapine titration, the patient at each consultation was reminded to contact the treating physician immediately if any kind of infection begun to develop and to give particular attention to flu-like complaints such as fever or sore throat and to other evidence of infection, which may have been indicative of neutropenia. She had cautious titration and close monitoring of her white blood cell and absolute neutrophil counts, which remained relatively stable, within the normal ranges.

At the Emergency Department, the patient was oriented, with a depressive mood. From her clinical examination, she was obese (body mass index 32 kg/m^2^) and afebrile, and her blood pressure was 95/65 mm Hg, while her pulse was 95 beats per minute and regular. Chest examination and heart and breath sounds were normal. There was no hepatosplenomegaly, lymphadenopathy, abdominal tenderness, or mass. She presented with swelling, pain, warmth, and redness in the involved right leg, and she had difficulty walking. She had no weakness or sensory deficits. The patient also presented a rash on her legs, which was a confluent, nonblanching, erythematous, elevated patch consistent with a palpable purpura. The rash did not involve her palms or soles.

### 2.1. Investigations

The main investigations of the patient performed at Emergency Department and during hospitalization are presented at Table 1. The white cell count was 1.100 per cubic millimeter, with 0.0% (<500) neutrophils, 96.5% (1100) lymphocytes, 2.7% (0.0) monocytes, and 0.0% eosinophils. The hemoglobin level was 9.5 g per deciliter and the platelet count 264.000 per cubic millimeter. The C-reactive protein was 238.6 mg per liter. Erythrocyte sedimentation rate was 97 mm/1 h. D-Dimers were 10.29 mg per deciliter. Thyroid hormones and tumor markers were within normal limits. Further serologic tests, that is, hepatitis B, hepatitis C, Coombs test, anticardiolipin, anti-DNA, antinuclear, proteinase 3, and antineutrophil cytoplasmic antibodies (PR3-ANCA and cANCA), were negative. However, perinuclear antineutrophil cytoplasmic antibodies (pANCA) were positive and antineutrophil cytoplasmic antibodies against myeloperoxidase (MPO-ANCA) were increased.

**Figure 1:**
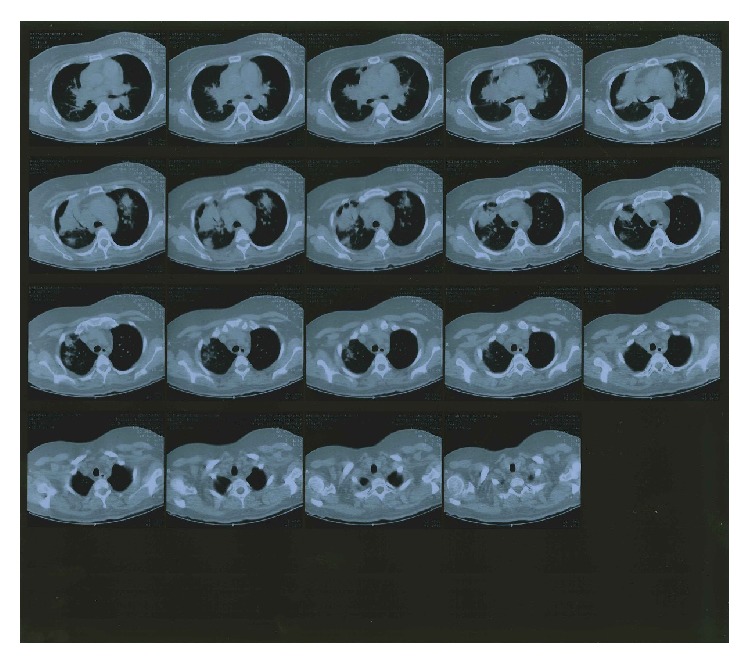
Acute pneumonia caused by* Streptococcus pneumoniae* alone in a 33-year-old female, 6 days after the onset of fever, cough, and dyspnoea. Transverse high-resolution CT image at the level of the right upper lobe shows consolidation, centrilobular nodules, and bronchial wall thickening.

The patient underwent compression ultrasonography and pulse-wave Doppler sonography which diagnosed thrombosis of the common femoral and right proximal iliac vein. Chest X-ray as well as high-resolution CT was performed which revealed ground-glass opacification and positive pneumobronchogram (Figure 1).* Streptococcus pneumoniae* was isolated from blood cultures collected from different peripheral vein sites from separate venipunctures and from sputter specimens.* Legionella pneumophila* urine antigen test was negative.

A punch biopsy of her skin revealed perivascular neutrophilic infiltrate with extravasation of red blood cells, suggestive of early leukocytoclastic vasculitis.

Flow cytometry performed revealed agranulocytosis and neutropenia (neutrophils <50 per cubic millimeter), lymphocytopenia, with low concentrations of T4, T8, B, and natural killer cells, eosinopenia, and monocytopenia. It also detected polyclonal B lymphocytes and absence of blasts or prodromal cells of the granulocyte series.

### 2.2. Treatment

Clozapine was immediately discontinued. IV empirical antibiotic therapy with an antipseudomonal *β*-lactam agent, that is, piperacillin-tazobactam 4.5 gr t.i.d., was initiated. To the initial regimen aminoglycoside, that is, amikacin 1 gr b.i.d., was then added, together with vancomycin 1 gr b.i.d. for management of pneumonia and associated complications, that is, hypotension as well as suspected antimicrobial resistance. As the patient remained hemodynamically unstable, her antimicrobial regimen was broadened to include coverage for fungi, that is, fluconazole 100 mg b.i.d. [17].

**Figure 2:**
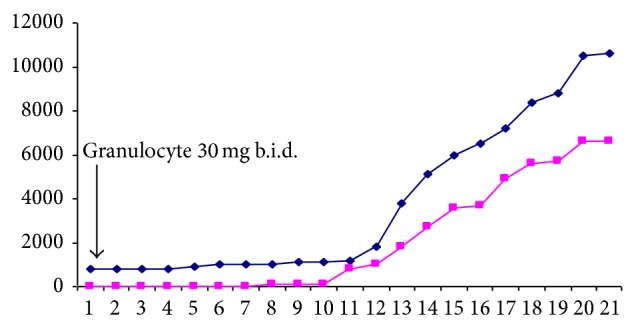
The course of the patient's white blood cell and leukocyte count during granulocyte treatment; blue line: white blood cell count, pink line: leukocyte cell count; b.i.d.: twice a day.

**Figure 3:**
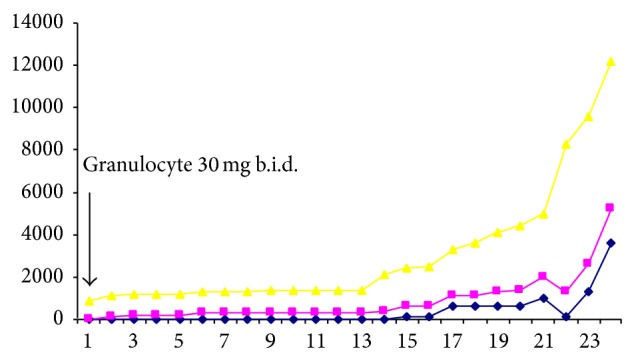
The course of the patient's eosinophils (blue line), basophiles (pink line), and monocytes (yellow line) white blood cell count during granulocyte treatment; b.i.d.: twice a day.

G-CSF 30 mg b.i.d. was subcutaneously initiated on day 1. During and after granulocyte treatment, her neutrophil count rose, her clinical condition improved, and her lungs cleared. The numbers of neutrophils and monocytes increased and eosinophils appeared in the circulation.  Figure 2 demonstrates the course of the patient's white blood and leukocyte count during granulocyte treatment. Moreover, Figure 3 demonstrates the course of the patient's eosinophils, basophiles, and monocytes white blood cell count.

The diagnosis of allergic vasculitis was made because of the patient's age, recent medication adjustments, the isolated skin involvement, the laboratory findings, and the results of the skin biopsy. She was managed conservatively without steroids and clozapine withdrawal was closely related with disappearance of pANCA and MPO-ANCA.

### 2.3. Outcome and Follow-Up

Olanzapine 10 mg was the alternative therapy introduced to our patient in order to stabilize her symptoms during treatment and then increased to 20 mg without effectiveness. Olanzapine was started 17 days after discontinuation of clozapine and when white blood cell count was within normal limits (4 ∗ 10^3^/*μ*L) with 47.5% (2000) neutrophils. As severe and prolonged relapse followed clozapine discontinuation, clozapine rechallenge was considered and successfully titrated with careful monitoring. No concurrent treatment with lithium or G-CSF was needed. The patient remains stabilized with no adverse effect reported till today.

## 3. Discussion

We presented a case of late clozapine-induced absolute neutropenia and agranulocytosis in a patient with BD treated with clozapine for 2 years. She was complicated by* Streptococcus pneumonia*, sepsis, venous thromboembolism, and allergic vasculitis.

With clozapine treatment, the risk of agranulocytosis is greatest in the first 18 weeks and is significantly reduced after 1 year, with its incidence falling to 0.07% in the second year [18]. Although risk of agranulocytosis decreases with time, in accordance with our study, some cases are reported after the second year of continued therapy. The incidence of agranulocytosis is reversible in the vast majority of cases, once clozapine is withdrawn promptly [18]. In our patient, immediate discontinuation of clozapine upon diagnosis, prompt initiation of antibiotic therapy, and G-CSF titration managed the early increase in the neutrophil count and the improvement of the patient's clinical presentation.

Clozapine can induce two clinically distinct types of neutropenia [19]. The first type is a mild to moderate neutropenia (neutrophil count below 1.5 ∗ 10^3^/*μ*L but not lower than 0.5 ∗ 10^3^/*μ*L) which occurs in 1.8% of treated patients. When clozapine is discontinued, recovery is rapid (2–8 days). The second type of neutropenia is more severe with a neutrophil count below 0.5 ∗ 10^3^/*μ*L (agranulocytosis) and an incidence of 0.78%. In the second type, as in the present case, even if clozapine is stopped when the neutrophil count is just below 1.5 ∗ 10^3^/*μ*L, agranulocytosis develops in some patients usually within 2–5 days and generally lasting for 14–21 days. In such patients monitoring allows the early detection and treatment but not the prevention of neutrophil suppression [19].

The pathophysiologic mechanisms that produce clozapine-induced agranulocytosis are not well understood. There is an age-related increase in risk of 53% per decade, with adolescents having the lowest risk of agranulocytosis and the greatest likelihood of remaining on clozapine [20]. Additionally, some authors have suggested an increased incidence in women [11]. Occurrence of severe neutropenia is not dose-dependent [12], thus suggesting that a direct toxic effect on the hematopoietic bone marrow is unlikely. Recent studies suggested a genetic vulnerability for clozapine-induced agranulocytosis [21, 22]. Further evidence suggests that agranulocytosis associated with clozapine is an idiosyncratic (type B) reaction and may be immune-mediated or involve a toxic mechanism or both [23].

Although, in clinical practice, the discontinuation of clozapine is frequently followed by worsening of psychotic symptoms, functional status, and quality of life [24–26], treatment with clozapine is contraindicated in patients with history of clozapine-induced agranulocytosis or severe neutropenia and in patients with hypersensitivity to this drug [24]. Therefore, advice regarding the risks of rechallenge is often sought by psychiatric care providers.

In accordance with our study, recent meta-analysis has challenged the clinical practice of discontinuing clozapine upon the development of agranulocytosis and stimulated further research in the pathophysiology and clinical consequences of a clozapine rechallenge after a white blood cell decline, especially in patients with complex symptomatology where no sufficient therapeutic results can be achieved with other pharmacological intervention than clozapine [24, 27].

In the majority of studies, patients rechallenged with clozapine following agranulocytosis usually (62%) do not experience a second episode of blood dyscrasia, although in some cases (38%) a second episode more severe with longer duration and in the agranulocytotic range may occur. However, none of the rechallenged patients included died. Risk-modifying strategies include the use of G-CSF, antibiotics, and antifungals as appropriate required. G-CSF seems to significantly decrease the length of agranulocytosis and of hospitalization and therefore should, at least in patients with absolute neutrophil count <500/mm^3^, be administered within 48 hours [2, 27, 28].

Coadministration of lithium with clozapine has also been suggested as a means of preventing neutropenia with a 94.5% success rate [29, 30], which was even higher than after administration of GCS-F [31, 32]. However, there are concerns that lithium might mask impending agranulocytosis, making the combination potentially dangerous [2]. Since our patient did not develop dyscrasia in the earlier phase of treatment, that is, the first 18 weeks of treatment, she was considered to be of low risk when rechallenged with clozapine.

Routine hematological monitoring for other medications associated with blood dyscrasia, such as valproic acid, phenothiazines, carbamazepine, olanzapine, ibuprofen, chlorpromazine, triamterene, quetiapine, clonazepam, and risperidone, should also be considered in individuals who previously developed blood dyscrasia during clozapine treatment, even if they have not yet had any hematological adverse effects with their new medication [18, 33–35]. It is possible that exposure to clozapine could sensitize the immune system in some fashion, making individuals susceptible to blood dyscrasias with other drug use in the future [36].

Frequent laboratory testing is required, that is, twice per week for at least 3 months, because the rechallenge-associated neutropenia or agranulocytosis occurs faster and is more severe than during the initial treatment with clozapine [33]. Time to rechallenge, speed of titration, and total dose have not been shown to influence the hematological outcome.

One of the agranulocytotic complications presented by our patient was venous thromboembolism. Venous thromboembolism has been associated with risk factors such as smoking, trauma, immobilization, surgery, pregnancy, use of combined oral contraceptives, malignant disorders, and certain cardiac and haemostatic disorders, including factor V Leiden mutation. In our patient, no such predisposing factors were identified. Although a conclusive causal relation has not been established between clozapine and venous thromboembolism, the absolute risk of death from pulmonary embolism is increased among clozapine users compared with other antipsychotic users of drugs in large epidemiological studies [37, 38].

In most of these studies an elevated risk of venous thromboembolism was observed for antipsychotic drugs, with the highest risk for clozapine, and the risk seems to be dose-related. The underlying biological mechanisms explaining the association between clozapine and venous thromboembolism are not well defined. Body weight gain, sedation, enhanced platelet aggregation, increased levels of antiphospholipid antibodies, hyperprolactinemia, and hyperhomocysteinemia are considered as incriminating factors [39, 40]. Although the risk of venous thromboembolism in psychotic disorders may also be related to the underlying disease, strong consideration should be given to discontinuation of clozapine in patients experiencing a venous thromboembolism episode. Therefore, it is essential that physicians and patients are aware that venous thromboembolism may be an adverse drug reaction to the antipsychotic treatment so the condition is identified early and treated appropriately.

Clozapine-induced allergic vasculitis is a rare but serious complication that should be added to the adverse reactions to clozapine therapy. To our knowledge, only one case of clozapine-induced allergic vasculitis has been previously reported to promote awareness and expedite diagnosis and treatment of this adverse reaction [41].

One of our study's limitations is that some of our patient's data may not be complete as we did not have complete record on concomitant or previous medications and illnesses experienced by the patient. Although adherence to the blood monitoring schedule is an indicator of compliance with therapy, this was also not verified directly. Finally, some data, excluding the hematological data, have not been verified at source.

## 4. Conclusions

Clozapine-induced absolute neutropenia and agranulocytosis are confirmed as a late adverse effect of clozapine therapy. Early detection of severe clozapine-induced absolute neutropenia and agranulocytosis enables the effective treatment of its complications, that is,* Streptococcus pneumonia*, sepsis, and venous thromboembolism. A causal relation between clozapine and venous thromboembolism is further suggested. Allergic vasculitis is reported as an atypical clozapine-induced adverse reaction.

## Figures and Tables

**Table 1:** Main investigations of the patient performed during hospitalization.

Laboratory test	Result	Normal range
White blood cell count	1100	4–10 ∗ 10^3^/*μ*L
Neutrophils	0.0	42.2–75.2%
Lymphocytes	96.5	20.5–51.1%
Monocytes	2.7	1.7–9.3%
Hematocrit	28.2	36–46%
Hemoglobin	9.5	12–14 g/dL
Platelets	264	140–440 ∗ 10^3^/*μ*L
Reticulocytes	0.0	0.1–1.5%
Prothrombin time	14.2	9.6–11.6 sec
Activated partial-thromboplastin time	33.35	26–39 sec
Serum sodium	135	135–144 mEq/L
Serum potassium	3.3	3.2–4.8 mEq/L
Blood urea nitrogen	11	15–43 mg/dL
Creatinine	0.6	0.6–1.1 mg/dL
Aspartate aminotransferase (AST)	29	5–34 IU/L
Alanine aminotransferase (ALT)	48	0–55 IU/L
Gamma-glutamyltransferase (*γ*GT)	65	9–36 IU/L
Lactate dehydrogenase	276	134–279 IU/L
Serum amylase	14	25–125 IU/L
Immunoglobulin (Ig) G	11.3	7–16 g/L
Immunoglobulin (Ig) A	1.75	0.8–4.5 g/L
Immunoglobulin (Ig) M	1.02	0.4–2.5 g/L
Complement component C3	1.32	0.8–1.5 g/L
Complement component C4	0.34	0.1–0.4 g/L
Rheumatoid factor	17.3	0–20 IU/mL
C-reactive protein (CRP)	238.6	<5 mg/L
Erythrocyte sedimentation rate	97	0–22 mm/Hour
D-dimers	10.29	<0.5 mg/L

## Abbreviations

ADHD: Attention-Deficit/Hyperactivity Disorder
BD: Bipolar disorder
b.i.d.: Twice a day
CT: Computerized tomography
DSM-IV: Diagnostic and Statistical Manual of Mental Disorders 4th Edition
G-CSF: Granulocyte-colony stimulating factor
MPO-ANCA: Antineutrophil cytoplasmic antibodies against myeloperoxidase
pANCA: Perinuclear antineutrophil cytoplasmic antibodies
PR3-ANCA: Proteinase 3 antineutrophil cytoplasmic antibodies
QD: Once a day
t.i.d.: Three times a day.
